# Schools and London Hospitals

**Published:** 1920-10-30

**Authors:** 


### Schools and London Hospitals.

Recently, at the London County Council, Mr. J. W. Gilbert, the Chairman, recalled that some months ago the Council suspended its regulations in order to enable an appeal to be made for the London Schools Hospital Fund throughout the London schools.

Former Records Surpassed.

He was now able to report the result of that appeal. Since it was issued last April, the London schools had raised no less a sum than £22,615 for the benefit of the London hospitals, made up as follows :

| | | | |
|---|---|---|---|
| Allocated for particular hospitals ... | £10,383 | 4 | 11 |
| Not specially allocated and to be handed to King Edward's Hospital Fund for London ... ... ... ... ... ... | 10,386 | 5 | 1 |
| Sent direct to particular hospitals ... | 1,846 | 8 | 9 |

In securing such a result the pupils of the London schools—including those not directly connected with the Council—had surpassed all their records in former efforts.

He felt sure that the Council and all interested in the beneficent work of the hospitals would wish to congratulate most heartily the pupils and students in the schools upon their enthusiasm and devotion in so good a cause, and to express sincere thanks to the Committee of teachers who initiated the appeal, to the Executive Committee of teachers who for many weeks past had given so much time and effort to its direction, to the teachers in the schools, to whose generous co-operation the success of the appeal had been so largely due, and to the members of the Council's administrative staff who had rendered such valuable assistance.

Certain facts would illustrate the far-reaching character of the London Schools Hospital Fund organisation. The amount realised represented an average contribution of sixpence from each pupil in the London schools. The work undertaken was not merely a formal collection in the schools. Some of the schools arranged football matches, cricket matches, bazaars, concerts, and various forms of entertainment for the benefit of the fund, the success of which was shown by the total amount realised; moreover, while the collections had amounted to £22,615, nothing had been charged against this sum except certain definite out-of-pocket expenses amounting altogether to less than £50.

The Sense of Social Service.

Whilst the Council and those interested in the work of the London schools would greatly rejoice at the substantial financial benefit which would accrue to the London hospitals, they would derive even greater pleasure from the practical evidence of the spirit of good will and the sense of social service existing in the schools which was indicated by this great charitable act.

